# Are the Effects of Oral and Vaginal Contraceptives on Bone Formation in Young Women Mediated via the Growth Hormone-IGF-I Axis?

**DOI:** 10.3389/fendo.2020.00334

**Published:** 2020-06-16

**Authors:** Heather C. M. Allaway, Madhusmita Misra, Emily A. Southmayd, Michael S. Stone, Connie M. Weaver, Dylan L. Petkus, Mary Jane De Souza

**Affiliations:** ^1^Department of Kinesiology, Pennsylvania State University, University Park, PA, United States; ^2^Division of Pediatric Endocrinology, Massachusetts General Hospital and Harvard Medical School, Boston, MA, United States; ^3^Department of Nutritional Science, Purdue University, West Lafayette, IN, United States

**Keywords:** oral contraception, contraceptive vaginal ring, insulin-like growth factor-I, procollagen type I N-terminal propeptide, IGF-I generation test

## Abstract

**Purpose:** Combined hormonal contraceptive therapy has been associated with negative bone mineral density outcomes that may be route-dependent [i.e., combined oral contraception (COC) vs. contraceptive vaginal ring (CVR)] and involve the hepatic growth hormone (GH)/insulin-like growth factor-I (IGF-I) axis. The objective of the pilot study was to assess the impact of route of contraceptive administration on IGF-I and procollagen type I N-terminal propeptide (PINP) responses to an IGF-I Generation Test. We hypothesized that the peak rise in IGF-I and PINP concentration and area under the curve (AUC) would be attenuated following COC, but not CVR, use.

**Methods:** Healthy, premenopausal women not taking hormonal contraception were recruited. Women were enrolled in the control group (*n* = 8) or randomly assigned to COC (*n* = 8) or CVR (*n* = 8) for two contraceptive cycles. IGF-I Generation Tests were used as a probe to stimulate IGF-I release and were completed during the pre-intervention and intervention phases. Serum IGF-I and PINP were measured during both IGF-I Generation Tests. The study was registered at ClinicalTrials.gov (NCT02367833).

**Results:** Compared to the pre-intervention phase, peak IGF-I concentration in response to the IGF-I Generation Test in the intervention phase was suppressed in the COC group (*p* < 0.001), but not the CVR or Control groups (*p* > 0.090). Additionally, compared to the pre-intervention phase, PINP AUC during the intervention phase was suppressed in both COC and CVR groups (*p* < 0.001), while no difference was observed in the control group (*p* = 0.980).

**Conclusion:** These data suggest that changes in recombinant human GH-stimulated hepatic IGF-I synthesis in response to combined hormonal contraception (CHC) use are dependent on route of CHC administration, while the influence on PINP is route-independent. Future research is needed to expand these results with larger randomized control trials in all age ranges of women who utilize hormonal contraception.

**Clinical Trial Registration:** www.ClinicalTrials.gov registration NCT02367833.

## Introduction

Since the first oral contraceptive pills were approved in the 1960s, combined hormonal contraceptives (CHCs) have been used by millions of women worldwide. The most popular CHCs include combined oral contraception (COC), the transdermal contraceptive patch (TDC), and the contraceptive vaginal ring (CVR) (1, 2). Between 2015 and 2017, ~65% of women aged 15–49 years in the United States were using some form of hormonal or non-hormonal contraception, of whom ~ 13% were using some form of COC (1).

Previous investigations have demonstrated conflicting data for the impact of past and/or current COC use on bone mineral density (BMD), including protection against low BMD (3, 4), decreased BMD or suboptimal BMD gain (0.5–1.5%) (5–8) or equivocal BMD response between COC users and non-users (8–12). Many differences in BMD observed between COC users and non-users were site specific (e.g., lumbar spine, total hip, etc.), ethinyl estradiol (EE) dose specific, and age specific (e.g., adolescent, young adult, perimenopausal). Reductions in bone formation [procollagen type I N-terminal propeptide (PINP)] and resorption [C-terminal cross-linked telopeptides of collagen I (CTx)] markers have been reported to occur more rapidly in COC users (30 μg and 15 μg EE) compared to non-users (6). In a study of oligoamenorrheic athletes, those randomized to COC use (30 μg EE and 150 μg desogestrel) experienced a decrease in PINP with no change in N-terminal cross-linked telopeptides of collagen I (NTx) over 12 months, while those randomized to a transdermal 17β-estradiol patch (with cyclic micronized progesterone) demonstrated a smaller reduction in PINP and no change in NTx (13, 14). Based on these conflicting findings, further efforts to understand the potentially detrimental effects of COC use on bone are critically important.

Negative effects of CHC use on BMD are likely associated with route of administration impacting the hepatic growth hormone (GH)-insulin-like growth factor-I (IGF-I) axis. Systemic IGF-I, predominantly synthesized in the liver in response to GH secretion (15, 16), provides an important stimulus for bone formation (17). However, COC use may result in reduced hepatic IGF-I synthesis in young women (18, 19), likely due to the “first pass effect,” by which EE metabolism in the liver decreases hepatic synthesis of IGF-I (20). Conversely, direct systemic absorption of EE with TDC and CVR use, which circumvents the hepatic portal circulation, may exert fewer negative effects on hepatic IGF-I synthesis (21, 22), and thus may be less detrimental to bone. However, results of such studies are not consistent (3, 18, 23).

A second mechanism potentially contributing to the observed reduction in IGF-I concentrations with COC use is a suppressed hepatic response to GH. An IGF-I Generation Test can be used to test hepatic responsiveness to GH stimulation by providing an exogenous GH stimulus and measuring the IGF-I response (24–26). The test has the potential to amplify subtle differences not otherwise detectable by simply assessing changes in fasting serum IGF-I concentrations. In postmenopausal women, oral estrogen therapy reduced recombinant human GH (rhGH)-stimulated peak IGF-I production by 20% (25, 26). In premenopausal women, peak IGF-I concentrations following rhGH administration were reduced by 36% in COC users (monophasic and triphasic 20–35 μg EE) compared with non-users (27). The IGF-I Generation Test has never been utilized prospectively in premenopausal women prior to and during CHC use, or to compare different modes of CHC administration.

To date, there have been no prospective studies that directly compare the impact of COC vs. CVR on the GH/IGF-I axis in healthy, young women. The purpose of this pilot study was to assess the effects of short-term COC and CVR use on hepatic IGF-I production and systemic PINP concentrations compared to a non-therapy control group. We hypothesized that compared to pre-intervention, basal IGF-I and PINP concentrations would be reduced following contraceptive therapy in the COC group compared to the CVR and Control groups, with no differences between CVR and Control groups. We also hypothesized that the IGF-I and PINP response to the IGF-I Generation Test (peak concentration and area under the curve) would be attenuated following contraceptive therapy in the COC group vs. the CVR and Control groups.

## Methods

### Experimental Design

**Figure 1:**
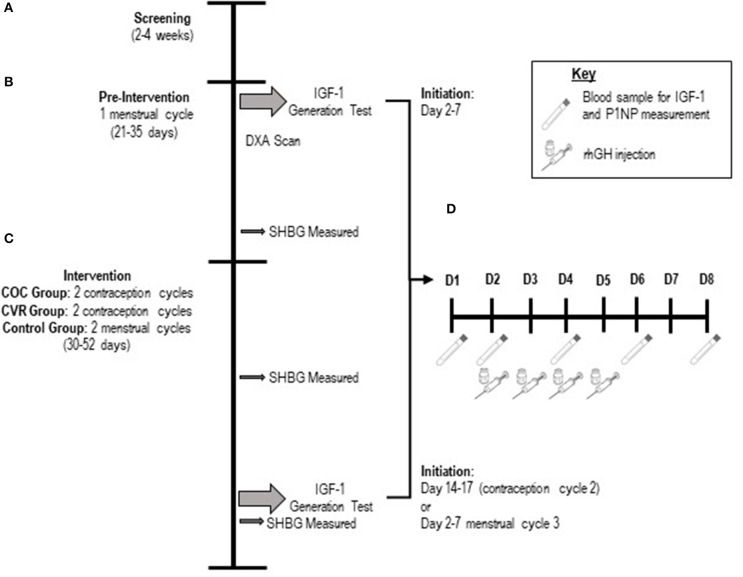
Schematic of the study design. **(A)** Screening lasted 2–4 weeks. **(B)** The pre-intervention phase was one menstrual cycle in duration. **(C)** The intervention phase was two contraception cycles or menstrual cycles in duration. **(D)** Schematic of the 8-day protocol for the IGF-I Generation Test. Participants had rhGH injections subcutaneously on days 2–5 of the 8-day protocol. Blood samples were taken from participants on days 1,2,4,6, and 8 of the 8-day protocol. COC, combined oral contraception; CVR, contraceptive vaginal ring; DXA, dual x-ray absorptiometry; rhGH, recombinant human growth hormone.

This pilot study was a prospective, open label, randomized controlled study examining the impact of CHC use on hepatic production of IGF-I in young women aged 18–30 years. The study was completed at two sites: the Pennsylvania State University (PSU; *n* = 17) and Purdue University (*n* = 9). Assessments were completed during an initial natural menstrual cycle (pre-intervention) and during the second contraceptive cycle (COC and CVR groups) or second natural menstrual cycle (Control group) of the intervention phase (Figure 1). The impact of COC vs. CVR use on hepatic IGF-I production and systemic PINP were assessed via measurements of basal IGF-I and PINP concentrations and serial IGF-I and PINP concentrations in response to an IGF-I Generation Test. The Institutional Review Boards of PSU and Purdue University approved the study protocol. All participants signed informed consent prior to initiating screening procedures in accordance with the Declaration of Helsinki. This study was registered at ClinicalTrials.gov (NCT02367833).

### Participants

Women not currently using hormonal contraceptives were recruited. Eligibility included: (1) age 18–30 years, (2) BMI 18–29 kg/m^2^, (3) non-smoking status, (4) naïve to hormonal contraceptives or not using hormonal contraceptives for at least 6 months prior to study entry, (5) not lactating, pregnant, or intending to become pregnant in the next 6 months, (6) no apparent metabolic, endocrine, musculoskeletal, or severe psychiatric disease, (7) if physically active, the primary mode was required to be weight bearing, (8) able to maintain current exercise training and diet, and remain weight stable (±2 kg) for the study duration, and (9) at least nine menses in the past 12 months. Participants were excluded if they: (1) regularly consumed large amounts of soy products or grapefruit, (2) had a diagnosis of liver or renal disease, (3) had any malabsorption or skeletal disorder, (4) had uncontrolled thyroid abnormalities, (5) chronically used non-steroidal anti-inflammatory drugs, (6) used medications known to have interactions with hormonal contraception, (7) had any contraindication for hormonal contraception use as proposed by the World Health Organization (28), or (8) were a Division 1 athlete on or off season (PSU site).

### Screening (Figure 1A)

Anthropometrics, to include height (to the nearest 0.1 cm) and weight (to the nearest 0.05 kg), were measured. Participants completed questionnaires to assess medical, menstrual, and exercise history, eating behaviors, and psychological health. A screening blood panel and a physical exam were performed to determine overall health. Participants age 22 and older were required to provide evidence of a normal PAP smear in the preceding 18 months.

### Participant Grouping Categories

Participants self-selected into the Control group (*n* = 8) or were randomized to use either a monophasic COC (30 μg EE and 150 μg desogestrel; Reclipsen Actavis plc, Parsippany-Troy Hills, NJ; *n* = 9) or CVR (15 μg EE and 120 μg etonogestrel; NuvaRing^TM^, Merck, Kenilworth, NJ; *n* = 9) for two contraceptive cycles. The selection of the COC was based on the similarity of the progestin type and dose to that in the CVR progestin, published data regarding the similarity of EE bioavailability between this COC at nadir and CVR (29), and the COC dose is commonly prescribed by physicians.

### Study Phases

The study had two testing phases: pre-intervention (Figure 1B) and intervention (Figure 1C).

#### Pre-intervention

The *pre-intervention phase* (Figure 1B) occurred during a natural menstrual cycle, with the onset of menstrual bleeding indicating day 1 and testing beginning between days 2–7 of the menstrual cycle. The pre-intervention phase lasted the duration of the menstrual cycle (~4 weeks, i.e., until the onset of the subsequent menstrual bleeding). Participants completed an IGF-I Generation Test, initiated calcium supplementation (up to 1,000 mg/d) based on estimated daily calcium intake [Brief Calcium Assessment Tool (30)], initiated vitamin D supplementation (800 IU/d), had a DXA scan to assess body composition, had weekly body weight measurements, completed a menstrual calendar, and completed a 7-day exercise training log. During week 3 of the pre-intervention phase, participants desiring contraception were randomized to either the COC or CVR treatment group and prepared to initiate assigned CHC therapy with the onset of the subsequent menses.

#### Intervention

The *intervention phase* (Figure 1C) began on the first day of the first *or* second natural menstrual cycle following the pre-intervention menstrual cycle. The intervention phase lasted for two consecutive natural menstrual cycles in the Control group and two consecutive contraception cycles in the COC and CVR groups (42 days). The duration of two contraception cycles was necessary to (1) ensure a minimal yet adequate intervention phase, (2) allow ample time to schedule all follow-up testing, and (3) ensure intervention testing occurred while COC or CVR therapy was not interrupted with a hormone-free interval (placebo pills in COC group or no ring in CVR group). For the first contraception cycle, CHC therapy was initiated on day 1 of the menstrual cycle (i.e., onset of menstrual bleeding) and the assigned CHC was used as marketed (21 days of active hormones and a 7-day hormone-free interval). For the second contraception cycle, therapy was initiated on the day immediately following the hormone-free interval of the first contraceptive cycle and used as marketed for the first 21 days, after which time a third round of CHC therapy was initiated, skipping the 7-days hormone-free interval. Throughout the intervention phase, body weight was measured weekly and participants continued calcium and vitamin D supplementation, completed a 7-day exercise training log, and maintained a menstrual/contraceptive therapy calendar.

At the end of the intervention phase, a second IGF-I Generation Test was performed (Figure 1C). The test was initiated between days 2–7 of the second natural menstrual cycle of the intervention phase for the Control group or between days 14–17 of the second contraception cycle for the COC and CVR groups. As noted above, participants did not utilize the hormone-free interval for the second contraception cycle and were provided with additional COC pills or a CVR to allow for continued use of the COC or CVR until the conclusion of the intervention phase IGF-I Generation Test. During the intervention phase IGF-I Generation Test, participants continued calcium and vitamin D supplementation, completed a 7-days exercise log and maintained a menstrual/contraceptive therapy calendar.

### IGF-I Generation Test

Participants underwent an IGF-I Generation Test during the pre-intervention (Figure 1B) and intervention (Figure 1C) phases to probe the activity of the GH-IGF-I axis. Briefly, GH produced by the pituitary stimulates the production of IGF-I, primarily in the liver, but also in other tissues throughout the body. The IGF-I Generation Test uses rhGH as a stimulus and measures the resultant IGF-I production via serial blood samples to assess hepatic responsiveness to GH. By dosing participants with a rhGH dose that is based on body weight, a standardized assessment of the hepatic response to the same relative GH stimulus in all study participants is possible. The IGF-I Generation Test was 8 days in duration, consisting of 4 rhGH injections given subcutaneously into the abdomen at a dose of 0.033 mg/kg/day (31) and 5 fasting blood draws (Figure 1D). The pre-intervention phase IGF-I Generation Test was initiated between days 2–7 of the pre-intervention phase menstrual cycle. The intervention phase IGF-I Generation Test was initiated between days 2–7 of the second menstrual cycle of the intervention phase for the Control group or between days 14–17 of the second contraception cycle for the COC and CVR groups. Participants were asked to refrain from alcohol and resistance exercise for the duration (8 days) of the IGF-I Generation Tests and to not exercise in the morning before any blood draw or injection.

On day 1 of the IGF-I Generation Tests, participants had a blood draw (12 h fasted overnight). On day 2, a urine pregnancy test was performed and body weight measured to the nearest 0.05 kg. If the urine pregnancy test was negative, participants had a blood draw (12 h fasted overnight) followed by rhGH injection 1 of 4 (Omnitrope Sandoz, Holzkirchen, Germany). On day 3, participants had rhGH injection 2 of 4. On day 4, participants had a blood draw (12 h fasted overnight) and rhGH injection 3 of 4. On day 5, participants had rhGH injection 4 of 4. Participants again had blood draws on days 6 and 8 (12 h fasted overnight). No testing occurred on day 7. All testing occurred at the same time for all days of each IGF-I Generation Test and all testing was completed before 0930 h.

IGF-I and PINP were assessed in each of the 5 blood samples collected during the pre-intervention and intervention IGF-I Generation Tests. Hormone concentrations measured before rhGH administration (day 1 and 2) were averaged to obtain each participant's *basal* hormone (IGF-I and PINP) concentration during the pre-intervention and intervention phases. The maximum hormone (IGF-I and PINP) concentration measured in response to the rhGH administration (day 4, 6, or 8) was termed “*peak*” and the value was corrected for basal hormone concentration (i.e., peak minus basal) as an indication of the increment rise in IGF-I or PINP in response to the rhGH stimulus. Area-under-the-curve (AUC) for IGF-I and PINP were calculated for the pre-intervention and intervention IGF-I Generation Tests using the basal and day 4, 6, and 8 concentrations with Kaleidagraph Software (Synergy Software, Reading, PA, USA).

### Dual Energy X-Ray Absorptiometry (DXA)

Participants had a total body DXA scan performed to assess body composition during the pre-intervention phase. Measurements at PSU were performed using a GE Lunar iDXA (enCORE 2008 software version 12.10.113). Measurements at Purdue University were performed using a GE Lunar iDXA (enCORE version 15 SP1). Due to the general descriptive nature of the body composition measurements in this study, scanners were not cross-calibrated.

### Compliance

Participant compliance was monitored via assessment of daily menstrual/contraceptive calendars, returned COC and CVR packaging, and SHBG measured in 12 h-fasted blood samples (32). Blood samples to assess compliance were obtained between day 10–22 of the pre-intervention phase menstrual cycle, between days 13–21 of the first menstrual/contraceptive cycle of the intervention phase, and between days 13–21 of the second contraceptive cycle of the intervention phase. The Control group did not have a sample taken during the second menstrual cycle of the intervention phase, as testing occurred during the follicular phase, not the luteal phase.

### Hormone Assessment

Serum total IGF-I, intact PINP, and SHBG concentrations were measured in duplicate using chemiluminescent immunometric assays [IDS-iSYS; Immunodiagnostic Systems Limited, Gaithersburg, MD (IGF-I and PINP); Immulite, Diagnostic Products Corporation, Los Angeles, CA (SHBG)]. Analytical sensitivity for the total IGF-I and PINP assays were 8.8 ng/mL and 2 ng/mL, respectively, and assay limits were 10–1,200 ng/mL and 2–230 ng/mL, respectively. The intra-assay and inter-assay coefficients of variation were 0.97 and 0.8% for total IGF-I and 3.0 and 5.3% for PINP. Analytical sensitivity for the SHBG assay was 0.2 nmol/L and the assay upper limit was 180 nmol/L. Samples measured above the assay upper limit were diluted and assayed again, with the reported concentration being the product of the dilution factor. The intra-assay coefficient of variation was 1.47%.

### Statistical Analysis

Analyses were performed with SAS (version 9.4, Cary NC). Data were assessed for normality and outliers prior to analysis. No differences (*p* > 0.190) in body composition variables were observed between study sites, justifying the pooling of subjects from both study sites for analyses. Differences between study groups were assessed with Proc Mixed (age, gynecologic age, pre-intervention and intervention IGF-I Generation Test body weight, average daily rhGH dose, and body composition variables) or Proc NPar1Way (BMI). Data with multiple observations per participant (hormone concentrations: basal, peak, AUC) were analyzed as repeated measures using Proc Mixed with Tukey-Kramer *post hoc* analyses, with pre-intervention and intervention study phases as the two time points. Study group, study phase, and study group^*^study phase interaction terms were included in the model as fixed effects. Repeated measures over time were modeled with ante-dependence models for IGF-I and PINP. SHBG compliance measures were assessed for each study phase (pre-intervention, intervention menstrual/contraceptive cycle one, and intervention contraceptive cycle two) independently utilizing Proc Mixed with Tukey-Kramer *post hoc* analysis. Data are presented as mean ± SEM. Using a sensitivity analysis, with 3 study groups, 24 participants, 2 measurements (pre-intervention and intervention phases), an alpha = 0.05, and adequate power (1-β = 0.80) we were able to detect an effect size of 0.35.

## Results

### Participant Flow and Demographics

**Figure 2:**
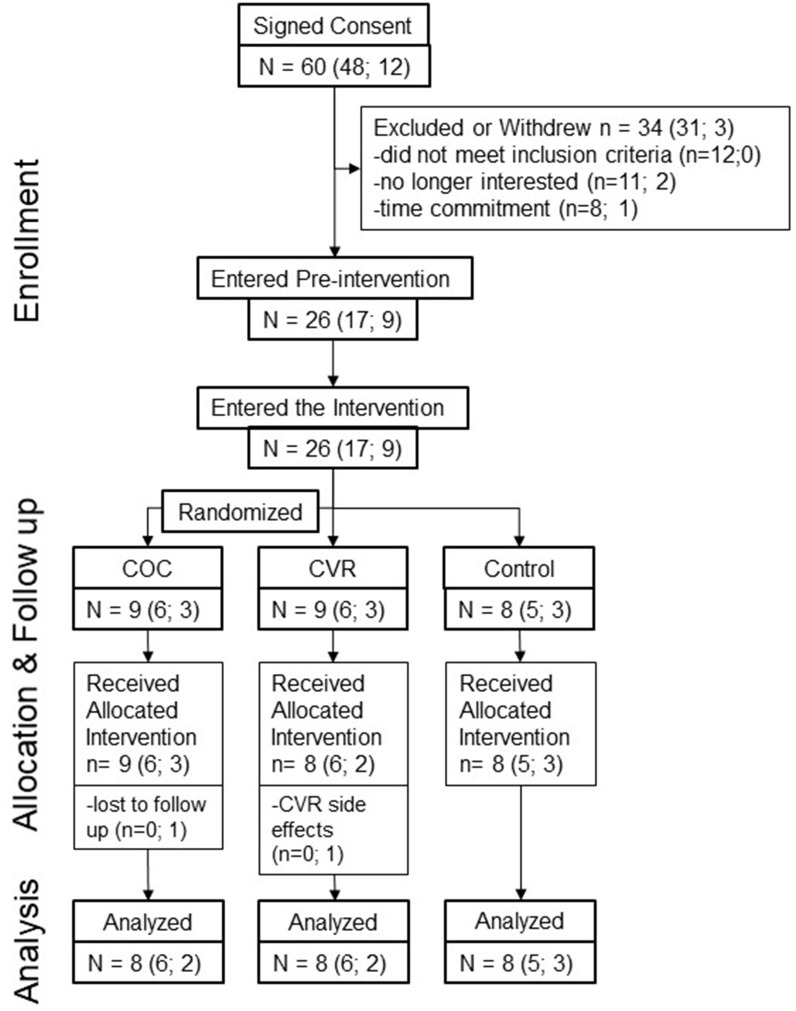
Progression of study participants through the pilot study. Total study N is provided as well as n per study site in brackets (PSU; Purdue). COC, combined oral contraception; CVR, contraceptive vaginal ring.

Of the 60 women who signed informed consent and were screened (Figure 2), 34 women withdrew (*n* = 22) or were found to meet study exclusion criteria during screening (*n* = 12). Thus, *n* = 26 participants entered and completed pre-intervention testing (*n* = 8 Control, *n* = 9 COC, *n* = 9 CVR). In the CVR group, three women experienced break-through bleeding; one withdrew due to this side effect. One participant in the COC group was lost to follow up on day 2 of the IGF-I Generation Test during the intervention. Thus, the intervention phase was completed by 24 women (*n* = 8 Control, *n* = 8 COC, *n* = 8 CVR). Analyses were performed on participants who completed all testing in the pre-intervention and intervention phases.

Participants in the COC, CVR, and Control groups did not differ with respect to age, gynecological age, screening BMI, body weight, body fat percentage, or lean body mass (*p* > 0.430) (Table 1). The average daily dose of rhGH did not differ among COC, CVR, and Control groups during the pre-intervention (*p* = 0.929) or intervention (*p* = 0.966) IGF-I Generation Tests.

**Table 1:** Participant demographic and body composition characteristics by study group.

	**COC**	**CVR**	**Control**	
	***n* = 8**	***n* = 8**	***n* = 8**	***P*-value**
**Demographics**				
Age (y)	22.3 ± 1.3	23.1 ± 1.4	23.6 ± 1.0	0.728
Gynecologic age (y)	9.9 ± 1.6	10.6 ± 1.6	9.5 ± 1.2	0.865
BMI (kg/m^2^)	23.2 ± 1.3	22.5 ± 0.6	21.9 ± 1.2	0.435
Pre-intervention IGT Weight (kg)	60.3 ± 2.3	59.5 ± 2.2	58.7 ± 4.4	0.939
Intervention IGT weight (kg)	60.7 ± 2.4	59.5 ± 2.0	58.9 ± 4.7	0.929
**Body composition**				
Body fat (%)	32.3 ± 2.0	30.1 ± 1.4	29.4 ± 2.9	0.619
Lean mass (kg)	37.9 ± 1.7	39.3 ± 1.4	38.5 ± 2.0	0.846
**IGF-I generation test daily rhGH dose (mg/day)**				
Pre-intervention phase	2.0 ± 0.1	1.9 ± 0.1	1.9 ± 0.1	0.929
Intervention phase	2.0 ± 0.1	1.9 ± 0.1	1.9 ± 0.1	0.966

*Mean ± SEM; IGT, IGF-I Generation Test; COC, combined oral contraception; CVR, contraceptive vaginal ring*.

### Compliance

**Figure 3:**
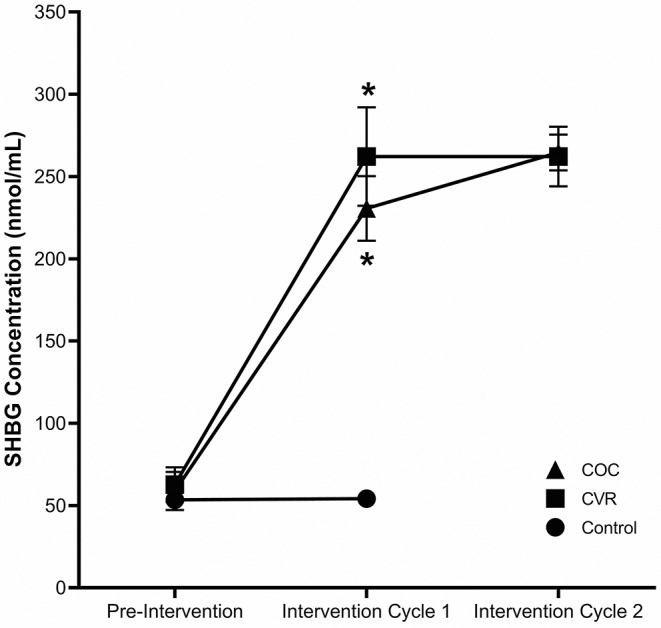
Compliance to study period and study group. SHBG concentrations for the COC (triangle), CVR (square), and control (circle), groups for pre-intervention, intervention cycle 1, and intervention cycle 2 compliance assessments. COC, combined oral contraception; CVR, contraceptive vaginal ring. * indicates difference from control group at same time point *p* < 0.05.

Review of the menstrual/contraception calendars indicated that COC users took the pill with consistent timing daily and participants in the COC and CVR groups appropriately initiated CHC use. Other than the aforementioned break-through bleeding in the CVR group, no severe menstrual symptoms or side effects of contraceptive use were noted. Analysis of SHBG concentrations in the three study groups (Figure 3) confirmed that participants were not on CHC during the pre-intervention phase (*p* = 0.783). During the first cycle of the intervention, SHBG levels indicated that the COC and CVR groups consistently used CHC, whereas the Control group continued not using CHC (*p* < 0.001). The two routes of CHC administration did not differ for the increase in SHBG levels during the first (*p* = 0.542) or second (*p* = 0.909) contraceptive cycles.

### Basal Hormone Concentrations

**Figure 4:**
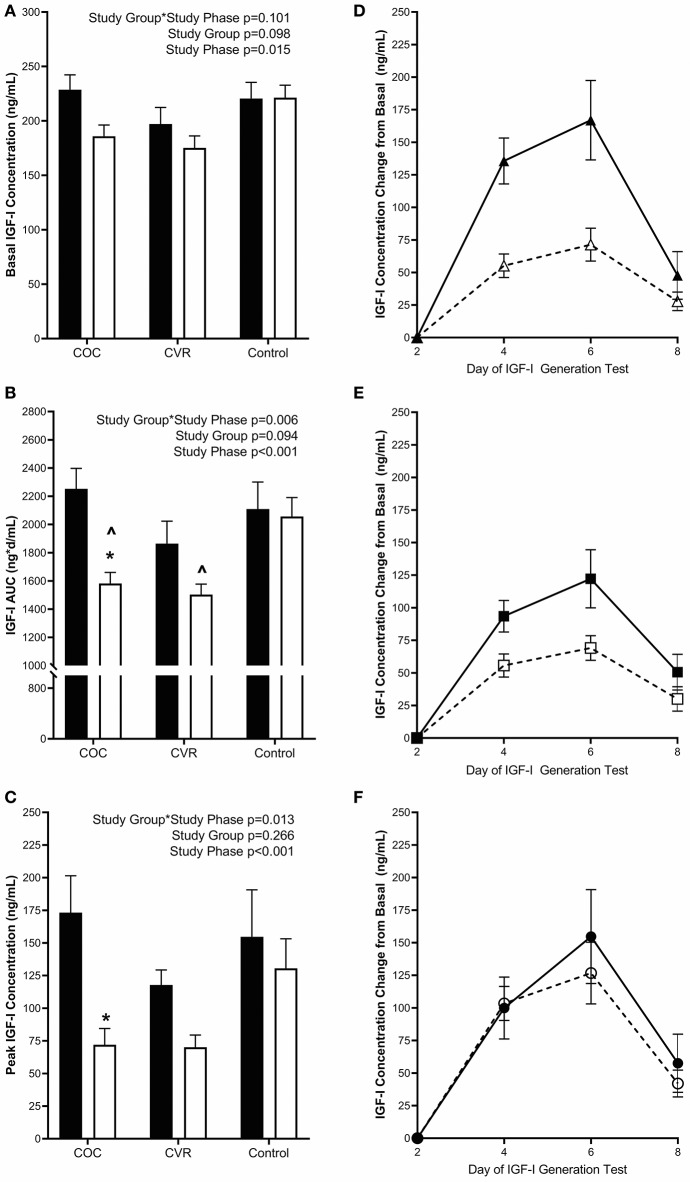
Pre-intervention (black bars) and intervention (open bars) IGF-I **(A)** basal concentrations and response to the IGF-I Generation Test measured as **(B)** AUC and **(C)** peak concentrations for the COC, CVR, and Control Groups. **(D–F)** Daily concentrations of IGF-I for pre-intervention (solid lines and filled symbols) and intervention (dashed lines and open symbols) phase IGF-I Generation Tests. **(D)** COC group (triangles); **(E)** CVR group (squares); **(F)** Control group (circles). Basal IGF-I concentrations were subtracted from day 4, 6, and 8 concentrations for each study group and study phase (pre-intervention and intervention. COC, combined oral contraception; CVR, contraceptive vaginal ring; IGF-I, insulin-like growth factor-I; AUC, area under the curve. * indicates difference from pre-intervention phase within study group *P* < 0.05. ^∧^ indicates difference from Control group within study phase *P* < 0.05.

Pre-intervention basal IGF-I concentrations (average of day 1 and 2 concentrations) did not differ among the COC, CVR, and Control groups (228.7 ± 13.6 vs. 197.1 ± 15.2 vs. 220.6 ± 14.8 ng/mL; *p* > 0.640) (Figure 4A). Basal IGF-I concentrations during the intervention did not differ among the COC, CVR, and Control groups (185.9 ± 10.3 vs. 175.2 ± 11.1 vs. 221.4 ± 11.5 ng/mL; *p* > 0.060). There was a main effect of study phase on basal IGF-I concentrations, such that an overall suppression of basal IGF-I concentrations was observed during the intervention compared to pre-intervention (*p* = 0.015).

**Figure 5:**
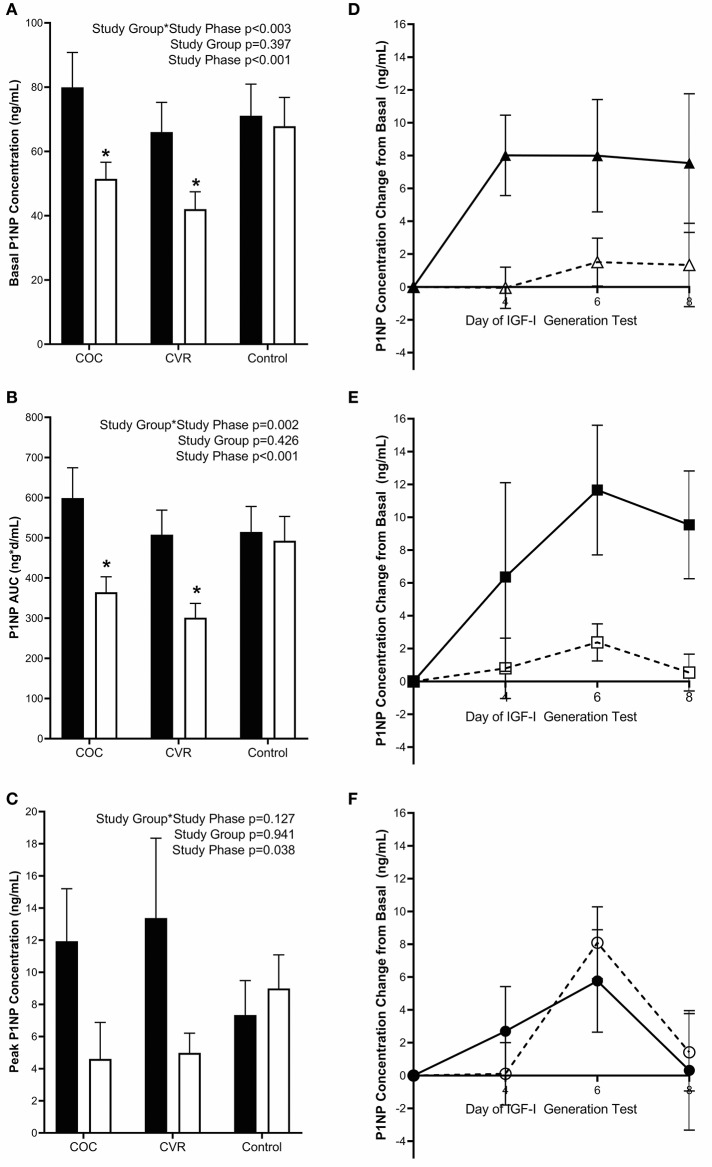
Pre-intervention (black bars) and intervention (open bars) PINP **(A)** basal concentrations and response to the IGF-I Generation Test measured as **(B)** AUC and **(C)** peak concentrations for the COC, CVR, and Control Groups. **(D–F)** Daily concentrations of PINP for pre-intervention (solid lines and filled symbols) and intervention (dashed lines and open symbols) phase IGF-I Generation Tests. **(D)** COC group (triangles); **(E)** CVR group (squares); **(F)** Control group (circles). Basal PINP concentrations were subtracted from day 4, 6, and 8 concentrations for each study group and study phase (pre-intervention and intervention). COC, combined oral contraception; CVR, contraceptive vaginal ring; PINP, procollagen type I N-terminal propeptide; AUC, area under the curve. * indicates difference from pre-intervention phase within study group *P* < 0.05. ^∧^ indicates difference from Control group within study phase *P* < 0.05.

Pre-intervention basal PINP concentrations did not differ among the COC, CVR, and Control groups (79.9 ± 10.9 vs. 66.0 ± 9.3 vs. 71.1 ± 9.9 ng/mL; *p* > 0.910) (Figure 5A). There was a study group^*^study phase interaction effect observed for basal PINP (*p* < 0.003). Basal PINP concentrations in the COC and CVR groups were reduced during the intervention compared to pre-intervention (*p* < 0.008), while no such effect on basal PINP concentrations was observed in the Control group (*p* = 0.983).

### IGF-I Generation Tests

Daily IGF-I (Figures 4D–F) and PINP (Figures 5D–F) concentrations during the IGF-I Generation Tests were graphed for each group (COC, CVR, and Control) and study phase (pre-intervention and intervention) as the difference from basal concentrations, which illustrates AUC.

#### Area Under the Curve

Pre-intervention IGF-I AUC did not differ among the COC, CVR, and Control groups (2252.6 ± 144.9 vs. 1863.7 ± 160.2 vs. 2109.2 ± 191.2 ng^*^d/mL; *p* > 0.570) (Figure 4B). IGF-I AUC was lower during the intervention in the COC and CVR groups compared to the Control group (1582.5 ± 78.7 vs. 1503.0 ± 74.4 vs. 2056.6 ± 134.2 ng^*^d/mL; *p* < 0.040), but did not differ between the COC and CVR groups (*p* = 0.992). There was a study group^*^study phase interaction effect observed for IGF-I AUC (*p* = 0.006); IGF-I AUC was reduced in the COC group during the intervention IGF-I Generation Test compared to the pre-intervention IGF-I Generation Test (*p* < 0.001). IGF-I AUC did not differ between pre-intervention and intervention IGF-I Generation Tests in the CVR or Control groups (*p* > 0.060).

Pre-intervention PINP AUC did not differ among the COC, CVR, and Control groups (599.1 ± 75.5 vs. 507.7 ± 61.4 vs. 514.8 ± 63.3 ng^*^d/mL; *p* > 0.920) (Figure 5B). There was study group^*^study phase interaction effect for PINP AUC (*p* = 0.002); PINP AUC was reduced in the COC and CVR groups during the intervention compared to pre-intervention IGF-I Generation Tests (*p* < 0.001). PINP AUC did not differ between the pre-intervention and intervention IGF-I Generation Tests in the Control group (*p* = 0.980).

#### Peak Response

Pre-intervention peak IGF-I concentrations (calculated as the highest concentration observed minus basal concentration) did not differ among the COC, CVR, and Control groups (173.3 ± 28.2 vs. 117.8 ± 11.5 vs. 154.7 ± 36.0 ng/mL; *p* > 0.130) (Figure 4C). There was a study group^*^study phase interaction effect observed for peak IGF-I concentrations (*p* = 0.013), peak IGF-I concentration decreased in the COC group from the pre-intervention to the intervention IGF-I Generation Test (*p* < 0.001). There were no significant differences between pre-intervention and intervention peak IGF-I concentrations in the CVR or Control groups (*p* > 0.090).

Pre-intervention peak PINP concentrations did not differ among the COC, CVR, and Control groups (11.93 ± 3.27 vs. 13.38 ± 4.97 vs. 7.34 ± 2.15 ng/mL; *p* > 0.810) (Figure 5C). Peak PINP concentrations during the intervention did not differ among the COC, CVR, and Control groups (4.61 ± 2.27 vs. 4.98 ± 1.22 vs. 8.99 ± 2.09 ng/mL; *p* > 0.595). There was a main effect of study phase, such that peak PINP concentration decreased from pre-intervention to intervention IGF-I Generation Tests in the study sample as a whole (*p* = 0.038).

## Discussion

This pilot study utilized an IGF-I Generation Test to garner a detailed understanding of the dynamics of bone trophic hormones before and during COC and CVR use in healthy, young women. Following COC use, hepatic responsiveness to rhGH stimulation was suppressed, as indicated by a reduction in IGF-I AUC (30%) and peak (58%) concentration compared to pre-intervention. IGF-I responsiveness was maintained following CVR use. The systemic response to COC and CVR was similar as indicated by reductions in basal PINP (36%) and PINP AUC (~ 40%) observed following COC, as well as CVR use. These novel findings suggest that while short-term CHC use suppresses bone formation markers, the specific mechanism by which this occurs is likely independent of route of administration.

The 58% suppression in peak IGF-I concentration observed following COC therapy is similar to previously reported responses to COC therapy (25, 27). During an extended rhGH stimulation study in premenopausal women, peak IGF-I concentrations were 52% above basal concentrations in non-users but only 16% above basal concentration in monophasic and triphasic COC users (20–35 μg EE) (27). In a cross-sectional study of postmenopausal women using oral estrogen therapy, a 58% suppression of peak IGF-I was observed compared to a no therapy control group (25). Similarly, in a 6-weeks crossover study of three different estrogen formulations compared to pre-therapy, peak IGF-I concentrations were suppressed by ~21% with oral estradiol valerate treatment (1 mg/12 h) but by only 13% with high-dose transdermal 17β-estradiol treatment (200 μg/d), while peak IGF-I with low-dose transdermal 17β-estradiol treatment (50 μg/d) did not differ from the pre-therapy test (26). Our observations, in concert with reports in pre- and postmenopausal women, indicate that hepatic capacity to respond to GH stimulation is possibly dependent upon the route of estrogen administration.

Our findings of reduced hepatic responsiveness to rhGH stimulation following short-term COC and CVR use likely have downstream implications for bone turnover, and ultimately BMD with continued use. IGF-I is widely regarded as bone anabolic, with IGF-I receptors located on osteoblasts (33). Strong positive correlations have been reported between IGF-I and multiple biochemical markers of bone formation, including PINP, osteocalcin, and bone specific alkaline phosphatase (BSAP) (34, 35). We observed a 36% reduction in basal PINP concentrations and a 40% reduction in the PINP AUC from the pre-intervention to intervention IGF-I Generation Tests in both the COC and CVR groups. Previously, investigators have reported suppression of PINP/PICP (3, 13, 14, 36), osteocalcin (7, 27), and BSAP (7, 27, 36) in COC and/or CVR users compared to non-users. However, such reports are contrasted by reports of no significant differences in PINP/PICP (27), BSAP (37), and osteocalcin (38, 39) in COC and CVR/TDC users compared to non-users. Though not measured in this study, suppression of bone resorption markers have been reported in cross-sectional and longitudinal analyses of COC (6, 7, 36–38) and CVR/TDC use (39), and should be assessed in future studies.

The types of progestins used in different CHC formulations may also contribute to the varying impact of CHC metabolism on bone. For instance, in women using COCs with 30 μg/d EE, a 30% reduction in basal IGF-I was observed in women using 2,000 μg/d dienogest (4th generation progestin), while a 12% reduction was observed women using 125 μg/d levonogestrel (2nd generation progestin) (40). The CHCs used in the present study were 3rd generation progestins (41). Desogestrel, the COC progestin, is rapidly and completely metabolized in cells of the liver and walls of the gut to its active metabolite etonogestrel, the CVR progestin (42). Thus, the progestin in the COC was subject to more extensive metabolism, which may have contributed to the greater reduction in IGF-I concentration observed compared to the CVR group. However, the consequences on PINP were similar following COC use and CVR use, which requires further investigation.

There were limitations of our study, including the inherent difference in monophasic COC and CVR available on the market, which resulted in the differences in the EE and progestin used in the investigation. Considerable effort was taken to choose the COC that would best match the daily hormone (both EE and progestin) bioavailability of the CVR available on the market (29). There are no COC available with etonogestrel and available desogestrel containing COC options have EE at 30 μg/day for 21 days, 20 μg/day for 21 days with 10 μg/day for 5 days or are triphasic. Therefore, the 30 μg/day of EE option was the closest to the CVR option and it is possible that the higher AUC of the EE dose noted in the literature (29) for our COC impacted the hepatic GH responsiveness more than accounted for by the first pass effect. Our inability to assess IGF binding proteins or a marker of bone resorption was limited by financial constraints. Importantly, our study provides prospective, preliminary data in a small sample of young women that successfully identified an avenue of research and the need and rationale for future investigations of both short-term and long-term use of CHC. Future investigations with a larger sample size in which more comprehensive profiles of the GH/IGF-I axis and bone turnover are evaluated at wider durations of use and a variety of age ranges will serve to enhance our understanding of bone health in response to CHC use.

In summary, our pilot study demonstrated that the administration of CHC impacts the dynamics of hormones involved in bone metabolism, though the effect depends on the dose of estrogen/progestin administered and the route of administration. While hepatic IGF-I responsiveness to GH stimulation was significantly blunted with COC use, bone formation, as indicated by PINP concentration, was suppressed following both COC and CVR use. Future investigations with larger number of participants are needed to better understand the complex, interrelated effects of CHC dose, route of administration, age at initiation, and duration of CHC use on bone turnover. The effects may have implications for peak bone mass accrual and/or future BMD and bone structural changes, which should be explored.

## Data Availability Statement

The raw data supporting the conclusions of this article will be made available by the authors, without undue reservation.

## Ethics Statement

The studies involving human participants were reviewed and approved by Institutional Review Boards of the Pennsylvania State University and Purdue University. The patients/participants provided their written informed consent to participate in this study.

## Author Contributions

HA was responsible for study approvals, study execution, data collection, data analysis and interpretation, and manuscript preparation and revision. MM was responsible for study design, data analysis and interpretation, and manuscript preparation and revision. ES was responsible for data collection, data analysis and interpretation, and manuscript preparation and revision. MS was responsible for study execution, data collection, and manuscript preparation and revision. CW was responsible for study design, study approvals, study execution, data collection, and manuscript preparation and revision. DP was responsible for study execution, data collection, and manuscript preparation and revision. MD was responsible for study design, study approvals, study execution, data analysis and interpretation, and manuscript preparation and revision.

## Conflict of Interest

The authors declare that the research was conducted in the absence of any commercial or financial relationships that could be construed as a potential conflict of interest.
